# A systematic approach to simultaneously evaluate safety, immunogenicity, and efficacy of novel tuberculosis vaccination strategies

**DOI:** 10.1126/sciadv.aaz1767

**Published:** 2020-03-04

**Authors:** Visai Muruganandah, Harindra D. Sathkumara, Saparna Pai, Catherine M. Rush, Roland Brosch, Ashley J. Waardenberg, Andreas Kupz

**Affiliations:** 1 Centre for Molecular Therapeutics, Australian Institute of Tropical Health and Medicine, James Cook University, Cairns & Townsville, Queensland, Australia.; 2 College of Medicine and Dentistry, James Cook University, Cairns & Townsville, Queensland, Australia.; 3 College of Public Health, Medical and Veterinary Sciences, James Cook University, Townsville, Queensland, Australia.; 4 Institut Pasteur, Unit for Integrated Mycobacterial Pathogenomics, Paris, France.; 5 Centre for Tropical Bioinformatics and Molecular Biology, James Cook University, Cairns & Townsville, Queensland, Australia.

## Abstract

Tuberculosis (TB) is the deadliest infectious disease worldwide. Bacille-Calmette-Guérin (BCG), the only licensed TB vaccine, affords variable protection against TB but remains the gold standard. BCG improvement is focused around three strategies: recombinant BCG strains, heterologous routes of administration, and booster vaccination. It is currently unknown whether combining these strategies is beneficial. The preclinical evaluation for new TB vaccines is heavily skewed toward immunogenicity and efficacy; however, safety and efficacy are the dominant considerations in human use. To facilitate stage gating of TB vaccines, we developed a simple empirical model to systematically rank vaccination strategies by integrating multiple measurements of safety, immunogenicity, and efficacy. We assessed 24 vaccination regimens, composed of three BCG strains and eight combinations of delivery. The model presented here highlights that mucosal booster vaccination may cause adverse outcomes and provides a much needed strategy to evaluate and rank data obtained from TB vaccine studies using different routes, strains, or animal models.

## Teaser

We provide an empirical model to evaluate and rank data obtained from preclinical tuberculosis vaccine studies.

## INTRODUCTION

Tuberculosis (TB) kills more people annually than any other infectious disease (*1*). Currently, live-attenuated Bacille-Calmette-Guérin (BCG) is the only licensed TB vaccine for human use. Although it has good ability to prevent extrapulmonary forms of TB in young children such as TB meningitis and miliary TB, the capacity of BCG to protect against pulmonary TB, the most common and transmissible form of the disease, is variable (*2*). Nonetheless, BCG continues to be widely administered intradermally in many TB endemic countries. To reach the World Health Organization’s 2035 The End TB Strategy goal of reducing the incidence of TB by 90% (*1*), and to stop the rise of multidrug-resistant strains of *Mycobacterium tuberculosis* (*Mtb*), the causative agent of TB, more efficacious vaccines are needed. Over the past two decades, several new TB vaccine candidates have been developed and are currently undergoing clinical testing. In addition to subunit vaccines composed of selected dominant *Mtb* antigens (*3*, *4*), live recombinant BCG strains and attenuated *Mtb* strains appear to be among the most promising vaccine candidates (*5*). It has also recently become apparent that the route of vaccine administration and whether the vaccine requires a booster application critically influence stage-gating decisions during the clinical development of novel TB vaccines. For example, a recently completed trial in South Africa that assessed the prevention of *Mtb* infection in neonatally BCG-vaccinated adolescents via booster immunization with the subunit vaccine candidate H4:IC31 or BCG, demonstrated a significant reduction in sustained QuantiFERON conversion by BCG revaccination (*4*). Although these results contrast with an earlier BCG revaccination trial in Brazil (*6*), they warrant further investigations into the importance of BCG booster application and whether this should occur by altering the route of administration or by engineering BCG to express additional antigens. While animal models can contribute to systematically answer these questions, there remains a discrepancy between preclinical studies, where immunogenicity and efficacy parameters are often prioritized, and clinical practice, where safety and efficacy are the dominant considerations.

*Mtb* has evolved various mechanisms to modulate and evade host immune functions such as phagolysosome fusion and antigen presentation to survive and replicate within macrophages. The ESX (type VII) secretion systems, while nonessential for bacterial growth, appear to be necessary for conferring virulence (*7*). Encoded within the region of difference 1 (RD1), early secreted antigenic target (ESAT)-6 secretion system-1 (ESX-1) is thought to play a role in phagosome disruption and activation of cytosolic pathogen recognition receptors. Genomic deletion of RD1 results in attenuation of *Mtb*, further implicating ESX-1 in virulence (*8*, *9*). All *Mycobacterium bovis* BCG strains, including those currently licensed for human use, lack RD1. Although an experimental strain of BCG reconstituted with an extended RD1 region from *Mtb* (BCG::RD1) has been demonstrated to reduce *Mtb* replication, it maintains proliferative capacity in lung and splenic tissues of immunocompromised mice, rendering this strain too virulent for human vaccine use (*10*). However, we have recently shown that ESX-1–mediated cytosolic contact of ESAT-6 is required to activate protective immune responses against TB (*11*). In this study, rapid secretion of interferon-γ (IFN-γ) only occurred when full-length ESAT-6 was secreted via ESX-1 but not when a C-terminal truncated version of ESAT-6, unable to reach the host cell cytosol, was expressed (BCG::RD1 ESAT-6 Δ92–95). Furthermore, improved protection against TB conferred by intravenous vaccination with BCG::RD1 at day 45 was lost when mice were vaccinated with BCG::RD1 ESAT-6 Δ92–95 (*11*). These findings support increasing evidence that initiation of cytosolic contact, as conferred by many recombinant BCG strains, improves protection (*12*).

In murine models, protection against TB generated through parenteral BCG administration relies on the induction of T helper 1 (T_H_1) CD4^+^ memory lymphocytes (*13*, *14*). However, many of the vaccine-induced lymphocytes lack the necessary mucosal-homing chemokine receptors to migrate into pulmonary tissue. This results in a delay in activation and recruitment of TB-specific effector (T_EM_) and central (T_CM_) memory lymphocytes to the lungs, allowing *Mtb* to infiltrate macrophages, proliferate, and establish chronic infection. In addition, a third type of memory T lymphocyte, largely CD69^+^CD103^+^ cells, called tissue-resident memory T lymphocytes (T_RM_), have recently been found to take permanent residence in nonlymphoid tissues. These cells are ideally situated in portals of entry, allowing them to mount timely recall responses unlike their circulating counterparts (*15*, *16*) and hence present as a possible cellular target for vaccination strategies.

Several studies have demonstrated that mucosal administration of BCG results in the generation of significant numbers of protective anti-TB airway T_RM_ (*17*–*19*). This suggests that the current protocol of administering vaccines parenterally needs to be reconsidered, with mucosal vaccination strategies appearing more efficacious (*20*). This also agrees with findings by Calmette and Guérin (*21*), published in 1931, that BCG vaccination administered via the oral route demonstrates good results. It has also been shown that T_RM_ can be generated in a number of peripheral barrier tissues (skin and mucosa) using the “prime and pull” booster vaccination method (*22*). In this strategy, memory lymphocytes are systemically primed using a traditional subcutaneous vaccination, followed by topical administration of inflammatory agents such as chemokines to the desired peripheral tissue. However, this strategy appears to be dependent on local antigen presence for generation of T_RM_ within the lung. Thus, a live vaccine capable of initially replicating in the host and maintaining antigenic stimulation may be more effective.

Despite these many efforts to develop more efficacious TB vaccines, intradermal BCG vaccination remains the gold standard. To facilitate preclinical decision-making and stage gating, here we used a systematic and comprehensive approach to investigate if a combination of recombinant BCG (rBCG) strains that express *Mtb*-specific antigens (BCG::RD1 and BCG::RD1 ESAT-6 Δ92–95), heterologous route vaccination [intratracheal (IT) and/or subcutaneous (SC)], and booster vaccination strategies can enhance protection against aerosol *Mtb* challenge and induce T_RM_. While C57BL/6 mice are the most commonly used preclinical small-animal model to study immunogenicity and efficacy in preclinical BCG development (representative of largely resistant healthy humans), SCID (severe combined immunodeficient) mice are traditionally used to assess the safety of live vaccines. To allow comprehensive safety evaluation within the C57BL/6 model itself, we have analyzed clinical, histological, and chemical pathology, such that safety can be evaluated simultaneously with immunogenicity studies. All parameters were compared to the current BCG protocol (represented by BCG::pYUB, a BCG strain containing a gene empty vector). An “empty vector” BCG control has been used to account for any effects that the vector may have on microbial fitness in vivo. In doing so, we developed an empirical model to integrate multiple measurements of safety, immunogenicity, and efficacy to rank each vaccination strategy. Using this approach, we find that (i) the expression of immunodominant antigens from the *Mtb* genome is the most effective way to enhance efficacy of BCG, (ii) that the mucosal administration of BCG booster doses is correlated with adverse safety outcomes, and (iii) that immunogenicity readouts do not predict efficacy. The model presented here provides a much needed strategy for the TB vaccine community to evaluate and rank data obtained from vaccine studies using different routes, strains, or animal models.

## RESULTS

**Fig. 1:**
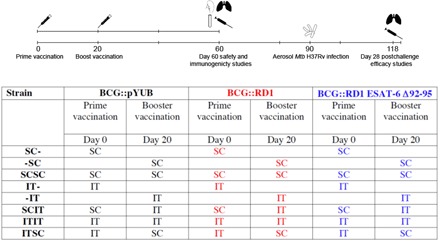
Schematic representation of vaccination/infection model. Six- to 8-week-old female naïve C57BL/6 mice were vaccinated via the SC or IT route or left unvaccinated. Twenty days later, animals received a booster vaccination via the same or alternate route or did not receive a booster. During the vaccination period, mice were weighed weekly and assigned daily a clinical health score. Sixty days after prime vaccination, some mice from each group were euthanized, and clearance of bacilli from the lungs and spleen, cellular immune responses in the lung interstitium and airways, lung histology, and serum cytokine/chemokine profiles were studied (vaccine safety and immunogenicity). At day 90 after prime vaccination, remaining mice were aerosol infected with *Mtb* H37Rv. At day 118, *Mtb* colony-forming units (CFU) from lung tissue was enumerated, and lung histopathology was analyzed (vaccine efficacy). Three recombinant strains of BCG were investigated: BCG::pYUB (standard BCG reconstituted with an empty control plasmid conferring resistance to hygromycin, serving as the control strain), BCG::RD1, and BCG::RD1 ESAT-6 Δ92–95. Together with unvaccinated mice, this resulted in a total of 25 experimental groups.

The experimental outline and experimental groups are shown in Fig. 1.

### Vaccine safety

Throughout the vaccine period, animal weight and signs of disease were monitored as clinical indicators of health. Sixty days after prime vaccination, vaccine clearance, lung histology, and serum cytokine profiles were analyzed. These parameters were selected as indicators of either lung or systemic health.

#### In vivo *persistence of BCG is dependent on the route of vaccination and strain characteristics*

**Fig. 2:**
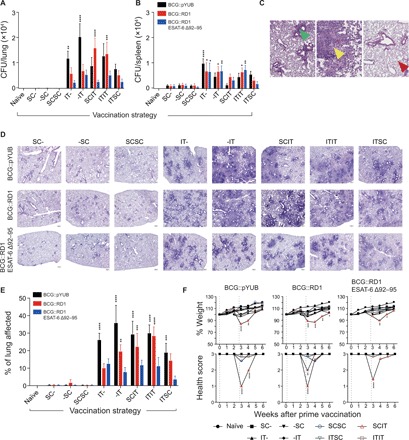
Vaccine safety. BCG CFU recovered from (**A**) lung tissue and (**B**) splenic tissue. (**C**) Representative images of iBALT-like structures (green arrowhead), generalized inflammation (yellow arrowhead), and normal lung tissue (red arrowhead) at ×40 magnification. (**D**) Representative H&E staining of lung sections from each experimental group at ×40 magnification. (**E**) Morphometric quantitation of histological changes presented as a percentage of the total lung area. (**F**) Weekly weight changes and health scores. Weight changes (top row) are presented as mean values from two pooled independent experiments (*n* = 6 to 13 mice per group). Health score results (bottom row) are presented as mean values from one representative experiment (*n* = 7 mice per group). (A to C) Presented as means ± SEM from two pooled independent experiments (*n* = 10 mice per group). The statistical significance of differences between each experimental group relative to unvaccinated controls is shown. *P < 0.05, **P < 0.01, ***P < 0.001, and ****P < 0.0001. The *P* values were determined using a one-way ANOVA followed by a Dunnett’s multiple comparison test.

The persistence of viable mycobacteria in the lung and splenic tissue of animals following vaccination was investigated as an indicator of local and systemic clearance of the vaccines. Sixty days following prime vaccination, viable bacilli were readily recovered from lung tissue of animals that were IT vaccinated, while minimal colony-forming units (CFU) were recovered from lung tissue of animals that received only SC vaccination(s) (Fig. 2A). Furthermore, IT vaccination resulted in higher CFU numbers in the splenic tissue when compared with SC vaccinated animals (Fig. 2B), indicating that IT delivery may allow for widespread dissemination of bacilli. Only BCG::pYUB and BCG::RD1 persisted in the lung tissue in significant amounts in some groups that received an IT vaccination. The number of bacilli recovered from the lung tissue of BCG::RD1 ESAT-6 Δ92–95–vaccinated mice was not significant, suggesting that this strain may be the most readily cleared from the lungs. The number of bacilli in lung tissues tends to reduce with time in all IT-vaccinated groups (Fig. 2A; -IT versus IT-). On the contrary, viable BCG::RD1 ESAT-6 Δ92–95 and BCG::RD1 remained in splenic tissue in significant amounts in more groups than BCG::pYUB.

#### IT vaccination induces inflammatory changes in the lung

To further assess the safety of each vaccination strategy, we assessed histological changes in lung tissue 60 days after the initial prime vaccination. In line with previous studies (*17*), hematoxylin and eosin (H&E) staining of lung sections from immunized animals revealed that IT BCG vaccination induced inflammatory changes in the lung parenchyma (Fig. 2C), including the presence of foamy macrophages, lymphocyte, and granulocyte infiltration. While some areas of tissue infiltration in close proximity to bronchioles resembled highly organized structures evocative of inducible bronchus-associated lymphoid tissue (iBALT) (Fig. 2C, green arrow head), other changes included edema and generalized foci of inflammation (Fig. 2C, yellow arrow head). Areas of consolidation and/or increased leukocyte infiltration were considered altered regardless of the pattern of inflammation (Fig. 2C, green and yellow arrow heads). Inflammation was almost completely absent in lung sections of animals that received only SC vaccination(s) and resembled the lung architecture of unvaccinated (naïve) animals (Fig. 2C, red arrowhead). IT vaccination, however, induced widespread inflammatory changes of heterogeneous patterns. Morphometric quantitation was performed, where the percentage of area affected by these changes was calculated (Fig. 2E). Animals vaccinated with BCG::pYUB and BCG::RD1 exhibited the greatest amounts of inflammation, while changes observed in animals vaccinated with BCG::RD1 ESAT-6 Δ92–95 did not reach significance (Fig. 2, D and E). Unexpectedly, all BCG::pYUB-immunized groups that received at least one IT vaccination demonstrated significant amounts of inflammation. BCG::pYUB also induced the greatest amount of inflammation across all vaccine strategies that involved IT vaccination. Comparison of groups that received a single IT vaccination at different time points suggested that these inflammatory changes were transient, as the percentage of lung involvement tended to reduce with time in BCG::pYUB- and BCG::RD1-vaccinated groups. Although this was not true for BCG::RD1 ESAT-6 Δ92–95–vaccinated mice, the changes induced in these animals were not significant at either time point, and thus, the slight increase in inflammatory change observed following BCG::RD1 ESAT-6 Δ92–95 vaccination may be negligible. It is, however, important to note that due to the large number of comparisons and multiple hypothesis testing in this study, individual comparisons among the entire group did not always pass our significance threshold, despite individual comparisons to unvaccinated mice across fewer groups showing statistical significance. Further investigation is needed regarding the different types of inflammation observed and whether pathological changes affect respiratory function or are protective structures that mediate immunity.

#### IT administration of booster vaccinations causes a transient state of illness

A single SC or IT vaccination did not cause any noticeable signs of disease, regardless of the vaccine strain administered. Body weights of these animals mirrored those of unvaccinated controls. However, animals that received a prime-boost vaccination strategy, where the booster was administered via the IT route (SCIT and ITIT; see table in Fig. 1), exhibited signs of illness following the booster dose. These animals suffered loss of weight and displayed clinical signs of illness including dyspnea, lethargy, and anorexia, 24 to 48 hours following the IT booster, with some animals euthanized for ethical reasons (three mice in the BCG::pYUB SCIT group and two mice in the BCG::RD1 SCIT group). The SCIT vaccination strategy had the greatest effect on health (Fig. 2F). Although this phenomenon was consistent among all vaccine strains investigated, animals vaccinated with BCG::pYUB developed the most severe clinical signs and suffered the greatest weight loss, while BCG::RD1-vaccinated animals were the slowest to regain weight. Despite this transient state of illness, animals appeared to recover as indicated by weight gain and returned to normal health scores by day 60. Collectively, these results imply that an IT booster vaccination with a live whole-organism vaccine administered 20 days after prime vaccination may be unsafe.

#### Prime-boost vaccinations with BCG::RD1 induce a prolonged systemic inflammatory profile

**Fig. 3:**
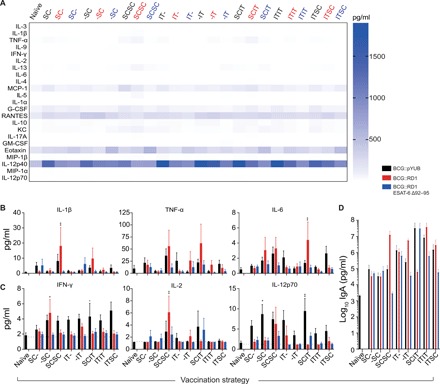
Systemic cytokine/chemokine profile and serum IgA levels before *Mtb* infection. (**A**) Serum cytokines/chemokines 60 days following prime vaccination presented in a heat map. (**B**) Serum levels of IL-1β, IL-6, and TNF-α used as measures of systemic inflammation for vaccine safety studies. (**C**) Serum levels of key T_H_1 cytokines (IFN-γ, IL-2, and IL-12p70). (B and C) Presented as means ± SEM from two pooled independent experiments (*n* = 10 mice per group). (**D**) Serum levels of IgA used as a measure of humoral immunity for immunogenicity studies (*n* = 3 mice per group). The statistical significance of differences between each experimental group relative to unvaccinated controls is shown. *P < 0.05 and **P < 0.01. The *P* values were determined using a one-way ANOVA followed by a Dunnett’s multiple comparison test.

To determine whether any of the vaccination strategies caused and maintained elevated levels of systemic cytokines, serum samples from vaccinated animals were analyzed for various chemokines and cytokines 60 days following prime vaccination (Fig. 3A). Across all vaccination strategies, a similar pattern was found. While proinflammatory cytokines are expected to increase immediately following vaccination, inflammation should subdue with time. Interleukin-1β (IL-1β), IL-6, and tumor necrosis factor–α (TNF-α) were used as markers of systemic inflammation due to their involvement in pathological hyperinflammatory states such as cytokine storm. None of the BCG::pYUB and BCG::RD1 ESAT-6 Δ92–95–vaccinated groups exhibited an increase in systemic levels of IL-1β, IL-6, and TNF-α relative to unvaccinated controls at day 60. However, animals immunized with BCG::RD1 via SCSC and SCIT strategies had significantly elevated levels of IL-1β and IL-6, respectively (Fig. 3B).

### Vaccine immunogenicity

To assess the immunogenicity of each vaccination strategy, serum cytokine/chemokine profiles, serum immunoglobulin levels, and airway and lung interstitial immune cell composition were analyzed 60 days after prime vaccination.

#### Parenteral vaccination is more likely to induce a T_H_1 cytokine milieu in the serum

T_H_1 effector responses have been implicated in the effective control of *Mtb* infection. Hence, key T_H_1 cytokine (IFN-γ, IL-2, and IL-12p70) levels in serum 60 days following prime vaccination were analyzed to determine which strategies established a T_H_1 profile. The only vaccination strategies that maintained significantly elevated levels of IFN-γ, IL-2, and/or IL-12p70 were BCG::RD1 -SC, BCG::RD1 SCSC as well as BCG::pYUB -SC and SCIT, respectively. None of the BCG::RD1 ESAT-6 Δ92–95–vaccinated groups maintained any of these cytokines at significant amounts (Fig. 3C).

#### IT vaccination increased serum immunoglobulin A levels

Immunoglobulin A (IgA) has been associated with enhanced protection against *Mtb* (*23*). To determine which strategy induced the greatest humoral response, total serum IgA concentrations were measured 60 days after prime vaccinations. Although vaccination appeared to generate a rise in antibody levels, none of the vaccine strategies induced statistically significant increases in IgA levels (Fig. 3D). Nonetheless, strategies that included at least one IT vaccination appeared to induce a trend toward stronger IgA response when compared with SC vaccination alone.

#### IT vaccination with BCG::RD1 induces significantly increased number of airway T_RM_

**Fig. 4:**
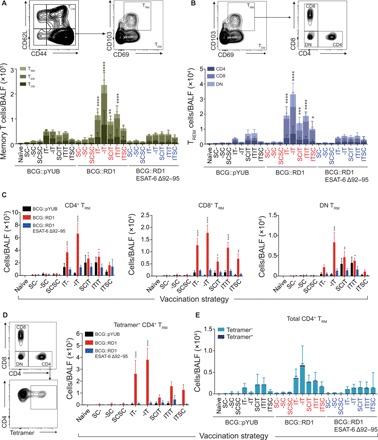
Airway immune cell profiles following vaccination. Sixty days following prime vaccination, animals were euthanized, and BALF was harvested for FACS analysis. (**A**) Representative FACS plots and enumeration of CD44^hi^ T lymphocyte subsets T_CM_, T_EM_, and T_RM_. Indicated significance is of total memory T lymphocyte numbers. (**B**) Representative FACS plots and quantification of CD4^+^CD8^−^ and CD8^+^CD4^−^ and CD4^−^CD8^−^ (DN) T_RM_. Indicated significance is of total T_RM_ numbers. (**C**) Total numbers of CD4^+^, CD8^+^, and DN T_RM_. (**D**) Representative FACS plots showing the gating strategy to enumerate tetramer^+^ cells and total numbers of tetramer^+^ T_RM_. (**E**) Proportion of tetramer^+^ and tetramer^−^ CD4^+^ T_RM_ are also shown. (A to D) Results are presented as means ± SEM from two pooled independent experiments (*n* = 5 to 10 mice per group). The statistical significance of differences between each experimental group relative to unvaccinated controls is shown. *P < 0.05, **P < 0.01, ***P < 0.001, and ****P < 0.0001. The *P* values were determined using a one-way ANOVA followed by a Dunnett’s multiple comparison test.

Since airway T_RM_ appears to be vital in protecting against *Mtb* (*18*) and interstitial T_RM_ is thought to replenish airway T_RM_ populations (*24*), phenotypic analysis of lymphocytes found in both compartments was performed. Sixty days following prime vaccination, bronchoalveolar lavage fluid (BALF) was harvested, and lungs were perfused with phosphate-buffered saline (PBS) through the right ventricle of the heart to eliminate lymphocytes that were not associated with pulmonary vasculature. Cells were isolated for flow cytometry to define and quantify the population of memory T lymphocytes generated in response to vaccination. IT vaccination generated increased numbers of both CD4^+^ and CD8^+^ T lymphocytes within the airways and interstitium when compared with vaccination strategies that only included SC administration(s). Many of these lymphocytes displayed a memory phenotype (CD44^hi^). Most of the memory T cells were peripheral tissue–homing lymphocytes (CD44^hi^CD62L^lo^), although substantial populations of central memory T lymphocytes (CD44^hi^CD62L^hi^) were also observed (Fig. 4A and fig. S1A). The characteristic coexpression of CD69 and CD103 within the CD44^hi^CD62L^lo^ population was used to differentiate between T_EM_ and T_RM_ (Fig. 4B and fig. S1B). When all 24 groups were statistically compared with unvaccinated mice, only strategies that included the IT administration of BCG::RD1 induced a significant population of airway T_RM_ (Fig. 4B). However, both BCG::pYUB and BCG::RD1 stimulated a significant T_RM_ response in the lung interstitium (fig. S1, A and B), while BCG::RD1 ESAT-6 Δ92–95 failed to do so in either compartment. Combined heterologous route vaccination strategies appeared to reduce the generation of interstitial T_RM_, as the T_RM_ population in the SCIT and ITSC groups did not reach significance.

Analysis of T_RM_ based on the expression of CD4 and CD8 revealed that CD8^+^ cells generally accounted for the largest proportion of T_RM_ established in airways following vaccination (Fig. 4C). A different pattern was observed in the interstitium, where BCG::pYUB-immunized mice developed a larger population of CD4^+^ T_RM_, while BCG::RD1-immunized animals developed a higher proportion of CD8^+^ T_RM_ (fig. S1C). Furthermore, double-negative T cells (CD4^−^CD8^−^) appeared to account for a smaller proportion of the T_RM_ population in interstitium compared with airways. Lymphocytes were also stained for the CD4 major histocompatibility complex (MHC) peptide tetramer derived from the immunodominant *Mtb* antigen ESAT-6 (QQWNFAGIEAAASA). Only groups vaccinated with a single dose of BCG::RD1 via the IT route established a significant population of tetramer^+^ cells in both the airways and lungs (Fig. 4D and fig. S1D). ITIT vaccination with BCG::RD1 appeared to markedly reduce tetramer^+^ cells in both compartments. In line with previously published data regarding lung T_RM_ retention (*25*), comparison of single IT vaccinations with BCG::RD1 at different time points (40 days, -IT; 60 days, IT-) also suggested that the population of tetramer^+^ T_RM_ induced by IT vaccination may not persist in the long term. From our data, it appears that a single IT vaccination generated the most dynamic and robust T lymphocyte response in the airways and lung interstitium at day 60. Vaccination with BCG::RD1 ESAT-6 Δ92–95 did not induce tetramer^+^ cells. It has been demonstrated that secretion of mycobacterial antigens is essential for their efficient presentation to CD4^+^ T cells (*26*, *27*). However, the situation in which biologically nonfunctional ESAT-6 molecules are secreted such as ESAT-6 Δ92–95 or the previously used ESAT-6 Δ84–95 constructs opens new interesting questions (*28*). Given that MHC-II peptide loading is not meant to involve cytosolic contact and that the C-terminal truncation of ESAT-6 does not overlap with the CD4^+^ T cell epitope tested, these results warrant further investigation into the role of ESAT-6–mediated cytosolic translocation for generation of ESAT-6–specific T cells.

### Vaccine efficacy

To determine the effect of each vaccination strategy on *Mtb* clearance and lung pathology, *Mtb* challenge experiments were conducted 90 days after prime vaccination.

#### All vaccination strategies and strains conferred similar levels of protection against Mtb burden 28 days after infection

**Fig. 5:**
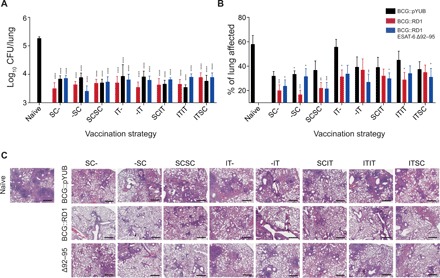
Vaccine-induced protection and pulmonary pathology following *Mtb* infection. (**A**) Twenty-eight days following *Mtb* infection, animals were sacrificed, and the lungs were assessed for numbers of viable *Mtb*. (**B**) H&E-stained lung sections were analyzed for histopathology. (**C**) Representative H&E staining of lung sections from each group at ×40 magnification. (A and B) Presented as means ± SEM from two pooled independent experiments. (A) *n* = 7 to 12 mice per group and (B) *n* = 6 to 8 mice per group. The statistical significance of differences between each experimental group relative to unvaccinated controls is shown. Scale bar, 600 μm. *P < 0.05, **P < 0.01, ***P < 0.001, and ****P < 0.0001. The *P* values were determined using a one-way ANOVA followed by a Dunnett’s multiple comparison test.

It has previously been reported that IT vaccination with BCG significantly reduces the bacterial burden at 45 days following *Mtb* challenge (at which point bacterial burden plateaus) when compared to SC vaccination (*17*). We investigated whether ESAT-6–expressing rBCG and/or heterologous route prime-boost vaccination strategies would induce increased protection early after *Mtb* infection. Ninety days after the priming vaccination, mice were aerogenically challenged with an ultralow to low dose of *Mtb* H37Rv (5 to 80 CFU) and were euthanized 28 days later. All vaccination strategies demonstrated significant reductions in *Mtb* CFU (1 to 1.5 log) when compared with unvaccinated controls (Fig. 5A). However, when compared holistically, there were no significant differences between experimental groups, suggesting that neither the rBCG strains tested nor the heterologous route prime-boost vaccination strategies led to increased protection at an earlier time point.

#### Vaccination with rBCG strains reduces lung pathology across vaccination strategies

Twenty-eight days following *Mtb* infection, histopathology analysis of H&E-stained lung sections and morphometric quantitation were performed (as described in the “Vaccine safety” section above). While some organized immune tissue changes reminiscent of tertiary lymphoid structures induced by IT vaccination were maintained, pathological changes such as pneumonia, granulomatous-like inflammation, and diffuse alveolar damage were prominent (Fig. 2E). Whether lung pathology in IT BCG–vaccinated groups are attributable to changes induced by the IT vaccination itself (Fig. 5, B and C) requires further investigation. Across all groups, animals that received BCG::RD1 or BCG::RD1 ESAT-6 Δ92–95 tended to suffer the least amount of lung pathology.

### Vaccine empirical integrated model

**Fig. 6:**
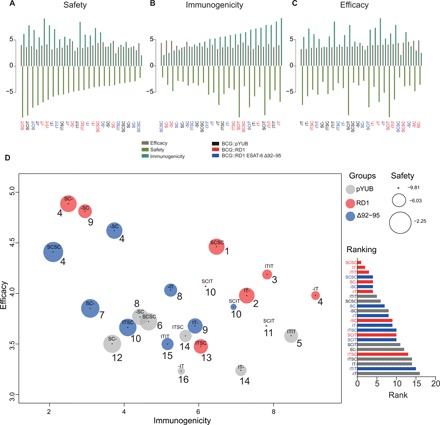
Vaccine empirical integrated model. Simultaneous comparison of safety, immunogenicity, and efficacy through integration of measurements into single measures of their summed effects (Eqs. 1 to 3 and Materials and Methods). (**A** to **C**) Vaccine strategies ordered from right to left based on increased safety, immunogenicity, or efficacy. (**D**) Vaccines plotted on overall performance of immunogenicity versus efficacy. The size of circles (D) is scaled to safety effect, where the safest strategy is represented by the largest circle. The overall ranking of each vaccine strategy is indicated by the number next to the circle and as a bar graph.

To simultaneously evaluate experimental parameters of vaccine safety, immunogenicity, efficacy, and overall performance, we developed an integrated approach, the vaccine empirical integrated model (VEIM) (Fig. 6 and VEIM methods; Eqs. 1 to 3). VEIM considers (i) efficacy as the combined effect of reduced *Mtb* CFU and lung pathology after challenge; (ii) immunogenicity as the combined effect of T_RM_ in BALF and lung, equally weighted levels of IL-2, IL-12p70, and IFN-γ, as well as IgA after vaccination; and (iii) safety as the combined effect of clinical score, weight reduction, lung and spleen bacterial burden, histopathological changes to the lung, and equally weighted levels of IL-1β, IL-6, and TNF-α after vaccination. For comparison, BCG::pYUB SC- was considered a representation of the current BCG strategy used clinically. Parenteral (SC- and SCSC) administration of the more virulent BCG::RD1 ESAT-6 Δ92–95 outperformed BCG::pYUB on safety, while BCG::RD1 SC- (previously deemed too unsafe for use in humans) ranked sixth (Fig. 6A). BCG::pYUB SC- appears to be one of the least efficacious vaccine strategies (Fig. 6C), while a single dose of BCG::RD1 obtained the greatest score for protection. Notably, vaccine strategies that ranked in the top half for efficacy were all rBCGs, while all BCG::pYUB-vaccinated groups ranked in the bottom half. This supports a model where the expression of key *Mtb* virulence factors is the most effective way of enhancing vaccine efficacy out of the strategies investigated. Furthermore, some of the most efficacious strategies ranked at the bottom for immunogenicity (Fig. 6B). Using our model, it appears that the most efficacious and safe vaccine strategies are single or double SC doses of BCG::RD1 or BCG::RD1 ESAT-6 Δ92–95 (Fig. 6D and table S2). When efficacy, safety, and immunogenicity were combined into a single rank (fig. S3A), our integrated data analysis indicates that two doses of BCG::RD1 given SC appear to be the most favorable strategy overall (Fig. 6D). To account for the fact that vaccine safety is always paramount and is more important than efficacy and immunogenicity, we also performed three alternate VEIM analyses, in which safety, immunogenicity, and efficacy were weighted differently to calculate the overall rankings (fig. S3B). In addition to equal ranking, the following situations were modeled: (i) safety as twice as important as efficacy and immunogenicity, (ii) both safety and efficacy as twice as important as immunogenicity, and (iii) efficacy in-between safety (as most important) and immunogenicity (as least important). In all scenarios BCG::RD1 SCSC remained the most favorable vaccination strategy, followed by single or double IT vaccinations with BCG::RD1. These findings suggest that BCG strains expressing *Mtb* antigens should be reevaluated in the context of mucosal and/or booster application and demonstrate that VEIM can be used in a versatile manner. VEIM also illustrates a disconnection between immunogenicity and protection of BCG strains more broadly (Fig. 6D), highlighting deficiencies in the understanding of true correlates of vaccine-induced protection against TB.

## DISCUSSION

It is widely accepted that studies evaluating novel TB vaccine strategies should include data regarding safety, immunogenicity, and efficacy. However, preclinical evaluation of new TB vaccines is biased toward immunogenicity and/or efficacy, and there remains no standard approach to integrating each of these parameters to determine how novel vaccine strategies perform overall in comparison to standard BCG. To address this, we used a systematic approach to evaluate and compare 24 vaccination regimens in the commonly used preclinical C57BL/6 mouse model. Upon completion of routinely performed data collection, we developed a model, VEIM, to rank vaccine strategies based on overall performance and simultaneous comparison of protection, immunogenicity, and safety. Our results show that expression of *Mtb*-specific immunodominant antigens is the most effective way to enhance efficacy of BCG and that mucosal BCG booster delivery correlates with adverse safety outcomes. VEIM also provides a tool to evaluate and rank data obtained from different TB vaccine studies.

SC administration of BCG provides some, but variable, protection against *Mtb* infection in murine models (*14*). Although T_H_1 immune responses have been implicated for protection, the exact immune response(s) that are responsible for reducing bacterial replication remain unknown (*29*). Following *Mtb* infection, T_RM_, a subset of memory T lymphocytes that take residence in peripheral tissue compartments has been observed in the lung tissue (*30*). It has also been shown that IT delivery of BCG enhances protection, correlating with increased T_RM_ numbers (*17*). Our study tested the hypothesis that heterologous route prime-boost vaccination strategies would enhance vaccine efficacy and T_RM_ numbers.

As previously demonstrated, CD4^+^ and CD8^+^ T lymphocyte numbers in the airways and lungs increased following IT vaccination, an increase not observed following standard parenteral vaccination. The statistically significant rise in T_RM_ numbers following IT administration of BCG::RD1 in airway and lung tissue appears to reduce within a 20-day period (40 days after vaccination compared with 60 days after vaccination). A similar decline in lung T_RM_ has been previously reported in other infection models, suggesting that low retention of immunocytes is an intrinsic feature of lung tissue (*25*, *31*). On the contrary, CD4^+^ T_RM_ was found 12 months after SC BCG vaccination with no measurable reduction in cell numbers in a murine model (*32*). However, it must be acknowledged that Bull *et al.* used intravascular staining and focused on lymphocytes found in the parenchyma rather than interstitium. In the present study, we did not use intravascular staining as it was logistically not feasible given the large numbers of experimental groups involved in our study. IT vaccination with BCG::pYUB only significantly increased T_RM_ numbers in lung tissue but not in the airway. Whether T_RM_ frequency in the airway or the lung tissue is more relevant to ultimate protective efficacy is currently unknown and requires further investigation. This also raises the question whether T_RM_ induction is influenced by characteristics of the vaccine strain. In addition to the genetic modification of BCG, there has been renewed interest in BCG vaccinations administered through other routes such as intravenous, oral, and skin scarification (*33*). Whether inclusion of these routes of administration in prime-boost strategies can provide better protection while remaining safe is worth exploring.

It was beyond the scope of this study to determine which strategy mediated best protection at later time points. However, it is widely acknowledged that mucosal vaccination provides better protection against pulmonary TB in multiple animal models. In murine models, this differential protection is particularly evident at 45 days after infection and up until at least 100 days after infection (*17*). Since mucosal booster strategies were unable to provide this enhanced protection at an earlier time point and caused adverse health outcomes, it may not be worth investigating the long-term protection mediated by these strategies, unless there are avenues to improve the safety of these strategies. The exact mechanisms of why animals vaccinated through SCIT and ITIT routes suffered weight loss and clinical illness are not clear, especially since these observations did not correlate with other parameters included in our study (although these additional parameters were only investigated after illness had subsided). While a single mucosal vaccination did not cause any adverse outcomes in our study, mucosal administration of BCG in macaques resulted in a slight (<3%) reduction in body weight 3 to 4 weeks after vaccination (*34*). Repeated exposure of *Mtb* was found to cause greater lung pathology than a single exposure to the same CFU dose in a rabbit model (*35*). Perhaps the prime-boost strategies used in our study mimic repeated exposure to mycobacteria, inducing pulmonary damage, weight loss, and overall health deterioration. Dosing regimens used in the present study were guided by previous studies. Prime and booster vaccinations were spaced 20 days apart, as the BCG-induced systemic T lymphocyte response takes 2 to 3 weeks to reach a substantial threshold. However, it is possible that the doses used were too high or too tightly administered in time. While IT vaccination is not applicable to a clinical setting, it was used in this study under a controlled experimental setting to elicit the mucosal immune response that is likely to occur in an aerosol delivery. Aerosol delivery was not used in this study as the number of bacilli delivered is highly variable, a separate problem for translatability of live aerosol vaccines. Although modifying dose and/or prime and booster timing may result in safer vaccination strategies, in clinical settings and human challenge models, it will be almost impossible to determine when natural exposure may have last occurred.

Aside from assessing prime-boost BCG vaccination strategies, we also investigated ESAT-6’s role in generating mycobacteria-specific T_RM_ and immunity. ESAT-6 is known to be a major T lymphocyte immunogen during *Mtb* infection, and lymphocyte restimulation with ESAT-6 is used as a diagnostic test for TB. In this study, we made use of an rBCG strain (BCG::RD1 ESAT-6 Δ92–95) designed to express ESX-1– specific virulence factors, including a C-terminal truncated ESAT-6. Specific mutations of ESAT-6 have been demonstrated to attenuate virulent mycobacterial species, potentially making them more suitable for use as vaccine strains (*36*). A previous study with BCG::RD1 ESAT-6 Δ92–95, however, has demonstrated that it is less capable of protecting against *Mtb* infection than strains that express unaltered ESAT-6 (*11*). Animals vaccinated with BCG::RD1 in our study produced a robust T lymphocyte response (including T_RM_), with a significant proportion being specific to QQWNFAGIEAAASA, an MHC-II ESAT-6 tetramer. Tetramer^+^ cells only accounted for a small proportion of the overall increase in T_RM_ numbers compared to BCG::pYUB- and BCG::RD1 ESAT-6 Δ92–95–vaccinated groups. This suggests that memory lymphocytes were also generated against other ESAT-6 epitopes and epitopes from other ESX-1 proteins. Although BCG::RD1 ESAT-6 Δ92–95 expresses the same repertoire of *Mtb*-specific antigens as BCG::RD1, it did not show the same capacity to stimulate an enhanced T cell response, instead generating memory cell numbers comparable to BCG lacking ESX-1 altogether. These data suggest that functional ESAT-6 is not only required for the noncognate production of IFN-γ in vivo but is also required for the generation of a robust T lymphocyte (including T_RM_) response against ESX-1–secreted effectors. These results may guide the development of future rBCG strains that have the capacity to express *Mtb* antigens.

To account for vector-related effects on in vivo persistence and fitness, we used BCG::pYUB rather than “standard” BCG. Unexpectedly, this empty vector BCG strain induced the greatest amount of inflammation across all vaccine strategies that involved IT vaccination. Although our statistical analyses and VEIM use pYUB as the control to subtract vector effects, this highlights the importance of determining vector-related effects in early preclinical studies. However, a limitation of this study is that this strain may have caused immunopathology in excess of standard BCG, leading to an overestimation of the relative safety of the recombinant strains. Nonetheless, the VEIM, which allows objective, simultaneous evaluation and ranking of various TB vaccination strategies used by other researchers in the field, is not affected by the use of BCG::pYUB. Furthermore, our findings that SC vaccination followed by an IT boost may be unsafe and that immunogenicity does not predict efficacy can be seen in all three BCG strains used and are, hence, not attributed to the use of BCG::pYUB.

The major benefit of using live vaccines is the stimulation of immune responses that most closely resemble those of natural infection. However, live vaccines are often contraindicated in immunocompromised individuals (those who need protection the most). Ideally, live vaccines should stimulate a strong memory response and be readily cleared. BCG has been shown to persist for up to 16 months after vaccination, allowing for a period of ongoing antigenic stimulation (*37*) but failing to confer a strong memory response in the pulmonary tissue. In our study, the numbers of recovered bacilli from the lungs of animals vaccinated with a single mucosal dose of either BCG::pYUB or BCG::RD1 ESAT-6 Δ92–95 approximately halved within a 20-day period, suggesting that these strains are being cleared from the lung tissue. Bacilli recovered from the lungs of BCG::RD1 IT–vaccinated animals, on the other hand, remained stable at 40 and 60 days after vaccination, supporting previous studies that this strain can persist in vivo (*38*). Although BCG::RD1 has been deemed too virulent for human use, our data suggest that a single mucosal or parenteral dose is safe (no loss in weight or health deterioration following vaccination) in immunocompetent animals, despite its persistence. Given that antigenic stimulation appears to be necessary for the retention of pulmonary T_RM_ and only BCG::RD1 was capable of eliciting a significant *Mtb*-specific T lymphocyte response in the pulmonary tissue, our study supports a model whereby the more “virulent” strains of attenuated mycobacteria need to be considered for use as vaccines against TB (*38*), if a benefit in protection can be demonstrated.

Currently, there is a lack of methods that permit simultaneous assessment of our experimental readouts of protection, immunogenicity, and safety mentioned above. Thus, we have developed VEIM to integrate routinely collected data from animal studies to rank and intuitively visualize vaccine strategies based on overall performance. Using this approach, two doses of BCG::RD1 administered parenterally rank as the most favorable vaccine strategy, characterized by being equally safe (if not safer) than control BCG::pYUB, immunogenic, and appearing more efficacious in protecting against disease. This supports a growing pool of evidence that live recombinant TB vaccines should express key *Mtb* virulence factors to enhance protection against pulmonary TB and that mucosal booster vaccination may cause adverse outcomes if administered at the peak of the primary immune response. Furthermore, our model demonstrates that despite selecting immunogenicity parameters based on current evidence, there does not appear to be a correlation between vaccine efficacy and immunogenicity. We find a small negative correlation (*R*^2^ = 0.15) driven by the low-immunogenic and high protection scores of BCG::RD1 (SC- or -SC) vaccination strategies. Together with previous reports (*39*), this finding highlights the limited understanding of the protective immune response against *Mtb* and correlates of protection in infection models.

Our model, to the best of our knowledge, is the first that attempts to simultaneously compare different vaccine strategies to aid the stage-gating pipeline of TB vaccine development. However, our model makes a number of assumptions, built on a priori biological premise, such as that immunogenicity, safety, and efficacy are additive effects, that different cytokines should have equal effect and are reliant on baseline control experiments to score improvements. Figure S3 shows that VEIM can be used in a versatile manner to include/exclude or to change the weighting of particular parameters in the model. This will be important to reach a consensus in the research community on how safety, efficacy, and immunogenicity should be weighted. Our model also does not consider genetic diversity, which is known to affect vaccine immunogenicity and efficacy. Thus, it would be beneficial to include additional parameters that account for genetic diversity by including studies from, for example, genetically diverse collaborative cross mice (*40*). Furthermore, it may be beneficial to include a wide range of parameters such as tertiary lymphoid structure generation, local antibody levels, mucosal-associated invariant T cells, and readouts of trained immunity (*41*, *42*) in future immunogenicity studies. Nonetheless, VEIM presents as an intuitive and readily extensible approach that allows for weighting of different parameters, numbers of replicates, and pairing of samples, and a simple assessment of multiple parameters that can be extended and applied more broadly to other vaccine/infection models. In particular, VEIM will be useful to dissect the relative importance of each included parameter within a vaccination challenge experiment. For example, the current gold standard for assessing the efficacy of TB vaccines in mouse models is the bacterial burden after *Mtb* challenge. However, as shown here, most current TB vaccine candidates only lead to relatively small CFU changes within a relatively narrow range. The comprehensive approach of the VEIM allows for these minor differences to be incorporated into an additive score, which allows unbiased ranking of each vaccination strategy.

The adaptability of VEIM will allow for constructive discussion within the TB research community to further fine-tune VEIM based on current evidence. The integration and visualization of multiple parameters have reinforced the need to further investigate the relationship between efficacy, immunogenicity, and safety. For example, what can we learn from the two relatively safe vaccination strategies of BCG::RD1(SC-) and BCG::pYUB (ITIT) that are highly protective, yet weakly immunogenic, or weakly protective, yet highly immunogenic to maximize overall vaccine performance?

In summary, our study provides evidence that parenteral administration of rBCG that expresses ESX-1 *Mtb* antigens enhances efficacy in protecting against TB while also maintaining a safety level comparable to control BCG::pYUB. Our study highlights potential concerns regarding the safety of mucosally administering live BCG vaccines to individuals who have previously been vaccinated, which should be considered in clinical studies and trials. We also developed a model, VEIM, which allows for evaluation of novel TB vaccine strategies to identify those that are most likely to confer better protection against TB while also being safe for human use. Approaches such as VEIM, which aim to standardize and integrate the analysis of new TB vaccine strategies and aim to identify those most worthy of further investigation, are invaluable tools for benchmarking performance against the current standard BCG delivery.

## MATERIALS AND METHODS

### Animals

Female C57BL/6 mice aged 6 to 8 weeks were bred and maintained under stringent specific pathogen–free conditions in the biosafety levels 2 (BCG vaccination experiments) and 3 (*Mtb* challenge experiments) animal facilities at the Australian Institute of Tropical Health and Medicine at James Cook University, Australia. Housing conditions included regulated ambient temperature (22°C) and lighting (12-hour light/12-hour dark cycle) with unlimited access to pelleted food and water in accordance with the Australian animal rights and regulations standards (equivalent to Institutional Animal Care and Use Committee guidelines). Animals were randomly allocated to experimental groups.

### Bacteria

BCG strains on the Pasteur background (provided by R. Brosch, Institut Pasteur, Paris) and *Mtb* H37Rv (sourced from the American Type Culture Collection) were cultured in Middlebrook 7H9 broth (BD Biosciences) supplemented with 0.2% glycerol, 0.05% Tween 80, 10% ADC enrichment (BD Biosciences), and relevant antibiotics. Mid-logarithmic [OD_600_ (optical density at 600 nm), 0.6 to 0.8] cultures were harvested, washed in PBS, and stored at −80°C until required. Before vaccine administration, frozen stock vials of vaccines were thawed, and the bacteria were centrifuged for 12 min at 3500 rpm. The resulting bacterial pellet was resuspended and diluted in PBS to the appropriate density as required for vaccination or infection (see below).

### Vaccinations and infection

C57BL/6 mice were immunized with commonly used doses of BCG: 2 × 10^5^ CFU for IT vaccination or with 1 × 10^6^ CFU for SC vaccination as shown in Fig. 1. Before IT immunizations, mice were anesthetized using a 1:16:160 xylazine-ketamine-PBS mixture administered intraperitoneally. Once anesthetized, mice were suspended from their upper incisors, and 20 μl of bacteria was inoculated into the oropharynx. The nostrils were occluded until the inoculum disappeared from the oropharynx. SC injections were performed into the base of the tail. These procedures were performed on day 0 (prime) and day 20 (boost) of the experimental timeline (Fig. 1). To determine protection conferred by vaccines, mice were challenged with an ultralow to low dose of *Mtb* H37Rv (5 to 80 CFU) via the aerosol route 90 days after vaccination using a Glas-Col inhalation exposure system. To determine the initial aerosol challenge dose, the lungs of five control mice were homogenized in 1 ml of PBS 0.05% (v/v) Tween 80, 24 hours after infection, and the entire volume of homogenized tissue was plated, incubated, and CFU was enumerated (refer to CFU enumeration below).

### Animal weight and health score

Animals from each group were weighed weekly using an electronic scale and were assigned a daily clinical health score based on several parameters including general appearance, behavior, respiration characteristics, and response to stimuli. Full health score criteria are provided in table S1. The lowest health score assigned during the course of the week was considered the overall health score for that 7-day period.

### Sample collection

Mice were euthanized using carbon dioxide asphyxiation or cervical dislocation. Blood for serum analysis was collected in serum separator tubes (BD Biosciences) from euthanized animals through cardiac puncture and allowed to sit for 4 hours. Coagulated blood was then centrifuged at 10,000 rpm for 6 min. Sera were stored at −20°C until analysis. To harvest the BALF, a small incision was made below the exposed larynx. Using an 18-gauge blunted needle, 1 ml of PBS was flushed into the lungs via the incision and aspirated. This was repeated three times to obtain a total of 3 ml of BALF per mouse. Organs were perfused with PBS through the left ventricle of the heart, and the lungs and spleen were harvested aseptically.

### CFU enumeration

For enumeration of BCG colonies after vaccination, perfused postcaval and inferior right lobes of the lung and spleen were homogenized in 1 ml of PBS 0.05% (v/v) Tween 80, and 100 μl was plated onto 10% OADC (BD Biosciences)–enriched Middlebrook 7H11 (BD Biosciences or Cell Biosciences) agar plates containing hygromycin B (50 μg/ml; Sigma-Aldrich). All strains of rBCG used were resistant to hygromycin B. For *Mtb* CFU enumeration, perfused right lungs were homogenized in 1 ml of PBS 0.05% (v/v) Tween 80, and 10-fold serial dilutions were performed (N, N^−1^, and N^−2^). Aliquots (100 μl) were plated onto 10% OADC–enriched Middlebrook 7H11 agar plates containing ampicillin (20 μg/ml), cycloheximide (50 μg/ml), and 2-thiophenecarboxylic acid hydride (2 μg/ml) (Sigma-Aldrich). 2-Thiophenecarboxylic acid inhibits the growth of BCG but not *Mtb*, thus allowing for enumeration of *Mtb* colonies only. In both instances, plates were sealed with parafilm, wrapped in aluminum foil, and aerobically incubated for 4 weeks at 37°C. Colonies were then counted, and dilution factors were accounted for in calculating the total bacterial burden of organs.

### Lung histology

Perfused left lung lobes from vaccinated unchallenged mice and unperfused left lung lobes from challenged mice were harvested and fixed in 10% neutral-buffered formalin for 24 hours and then stored in 80% ethanol at 4°C. Tissues were then processed and embedded in paraffin (HistoCore PEARL, Leica). Sections (4 μm) were cut and transferred onto glass slides (Snowcoat Clipped Corner Slides, Leica). The tissues were then deparaffinized using xylene and ethanol washes, rehydrated, and stained with Harris H&E using a Leica ST4020 Small Linear Stainer. Images were captured on a dissecting microscope at ×20 magnification. Histopathology/morphometric quantitation was performed using ImageJ (Fiji). The total area of lung tissue and areas of inflammatory change were calculated using the “Freehand selection” tool in ImageJ, which allows manual selection of areas of interest within an image.

### Cell isolation and preparation for flow cytometry

Airway luminal cells were harvested from BALF. Perfused right superior and middle lobes of the lungs were used for flow cytometry sample preparation. Lung-associated cells were prepared by cutting the tissue into small pieces using scissors. The diced tissue was incubated for 30 min at 37°C in RPMI 1640 medium with 10% fetal bovine serum (FBS), collagenase VIII (0.175 mg/ml), and collagenase D (0.075 mg/ml) (Sigma-Aldrich). Single-cell suspensions were prepared using mechanical dissociated through a 70-μm nylon mesh. To eliminate red blood cells, cell suspensions were incubated with EDTA (Sigma-Aldrich) for 5 min at room temperature, washed with fluorescence-activated cell sorting (FACS) buffer solution (PBS with 2% FBS), and placed on ice until analysis.

### Antibodies, tetramer, and flow cytometry

Identification of T cells was performed by using antibodies against CD3-A700 (clone 500 A2), NKp46-BV711 (clone 29A1.4), CD4-BUV395 (clone GK1.5), CD8-BV510 (clone), CD44-BV421 (clone IM7), CD62L–phycoerythrin (PE)–Cy7 (clone MEL-14), CD103–allophycocyanin (APC) (clone M290), and CD69-PE-CF594 (clone H1.2F3), all purchased from BD Biosciences. The I-A(b) *Mtb* ESAT-6 4–17 QQWNFAGIEAAASA tetramer (PE) was provided by the National Institutes of Health Tetramer Core Facility, USA. Fixable Viability Stain 780 (BD Biosciences) was used to exclude dead cells. Cells were incubated with viability stain for 10 min at room temperature and washed with FACS buffer solution. Samples were incubated with the tetramer (1:50 dilution) for 1 hour at room temperature and washed with FACS buffer solution. Cells were then incubated for 30 min with antibodies (1:200 dilution) on ice and washed with FACS buffer solution. Samples were resuspended in 150 μl of blank calibration particles (BD Biosciences) diluted in FACS buffer solution (1:74). Samples were analyzed using a FortessaX20 analyzer (BD Biosciences). Cells were enumerated using calibration particles 6.0 to 0.4 μm (BD Biosciences). CD3^+^NKp46^−^ cells were considered T lymphocytes. Memory cells were phenotyped as follows: CD44^hi^CD62L^hi^ (T_CM_), CD44^hi^CD62L^lo^CD103^−^CD69^−^/CD44^hi^CD62L^lo^CD103^−^CD69^+^/CD44^hi^CD62L^lo^CD103^+^CD69^−^ (T_EM_), and CD44^hi^CD62L^lo^CD103^+^CD69^+^ (T_RM_). The gating strategy for identifying tetramer^+^ CD4^+^ T_RM_ is shown in fig. S2.

### Serum multiplex analysis

Upon analysis, serum samples were thawed and prepared according to the Bio-Plex Pro Mouse Cytokine Standard 23-Plex Kit (Bio-Rad) specification. Serum samples were diluted in sample diluent (1:4), and beads and diluted samples were then incubated for 30 min at room temperature on a plate shaker at 850 rpm. The samples and beads were washed three times with wash buffer and then incubated with detection antibody as above. The samples and beads were rewashed three times and were incubated with streptavidin as previously. Following another three washes, the samples and beads were resuspended in assay buffer. Measurements were conducted on a MAGPIX instrument (Luminex).

### Serum immunoglobulin analysis

Upon analysis, serum samples were thawed and prepared according to the ProcartaPlex Mouse Antibody Isotyping Panel 2 7-Plex Kit. Serum samples were diluted in sample diluent (1:10,000), and bead and diluted samples were incubated for 60 min at room temperature on a plate shaker at 500 rpm. The samples and beads were washed. Detection antibody was added and allowed to incubate for 30 min at room temperature on a plate shaker at 500 rpm. The beads were rewashed and suspended in reading buffer. Measurements were conducted on a MAGPIX instrument (Luminex).

### Statistical analysis

Flow cytometry data were analyzed using FlowJo software version 10 (Treestar, CA). Statistical analysis was performed, and graphs were generated using GraphPad Prism version 7.0 (GraphPad software). One-way analysis of variance (ANOVA) followed by the Dunnett’s multiple comparison test was used with all experimental groups being compared against unvaccinated (naïve) controls, following testing for normal distribution of data by D’Agostino-Pearson omnibus normality test. Such a statistical method was chosen so that the groups with the greatest differences were highlighted. Any bias from the use of this statistical method and the underrepresentation of statistical differences between groups were addressed through the VEIM model, which uses raw data. No data points were excluded. *P* values less than 0.05 were considered significant. Heat maps and graphs for cytokine/chemokine data were prepared on GraphPad Prism version 7.0.

### Vaccine empirical integrated model

The VEIM was implemented in R version 3.5.1. Immunogenicity*_x_* (Eq. 1), safety*_x_* (Eq. 2), and efficacy*_x_* (Eq. 3) were developed as three additive models, where *x* was one of the 24 vaccination strategies tested and *u* was the unvaccinated control. Overall scores were ranked, and the rank sum was used to rank each vaccine according to four different weighting scenarios for safety, efficacy, and immunogenicity (full results are shown in table S2 and fig. S3B). Calculations were done using the following equations

$${\text{immunogenicity}}_{x}={BALF}_{x-u}+L_{x-u}+{IGA}_{x-u}+\frac{1}{3}(IL2_{x-u}+IL\;12p70_{x-u}+{IFN}_{x-u})$$ (1)

where *BALF*_*x*−*u*_, *L*_*x*−*u*_, and *IGA*_*x*−*u*_ represented the mean centered log_10_ *z* score difference of *x* versus *u*, and cytokines *IL2*_*x*−*u*_, *IL12p70*_*x*−*u*_, and *IFN*_*x*−*u*_ equally weighted as one cytokine score as the addition of rescaled to detection limit *z* score differences.

$${\text{safety}}_{x}=W_{x-u}+C_{x-u}+L_{x-u}+S_{x-u}+H_{x-u}+\frac{1}{3}(IL1b_{x-u}+IL6_{x-u}+{TNF}_{x-u})$$ (2)

where *W*_*x*−*u*_ represents the mean centered *z* score difference of the maximum difference of mean weight at any time point observed (weeks 1 to 6); *C*_*x*−*u*_ is the difference of clinical scores; *L*_*x*−*u*_, *S*_*x*−*u*_, and *H*_*x*−*u*_ represent difference of negative rescaled to zero z scores for lung BCG, spleen BCG, and histopathology, respectively; and cytokines *IL1b*_*x*−*u*_, *IL6*_*x*−*u*_, and *TNF*_*x*−*u*_ are equally weighted as one cytokine score as the addition of negative rescaled to detection limit *z* score differences

$${\text{efficacy}}_{x}=L_{x-u}+H_{x-u}$$ (3)

where *L*_*x*−*u*_ and *H*_*x*−*u*_ represent the negative *z* score difference of lung CFUs and histopathology, respectively.

### Study approval

All animal experiments were conducted according to the Australian National Health and Medical Research Council (NHMRC) guidelines and in accordance with requirements by the animal ethics committee of James Cook University. Institutional ethics approval (A2346) for the studies was granted by the James Cook University Animal Ethics Review Committee.

## Supplementary Material

http://advances.sciencemag.org/cgi/content/full/6/10/eaaz1767/DC1

Download PDF

A systematic approach to simultaneously evaluate safety, immunogenicity, and efficacy of novel tuberculosis vaccination strategies

## SUPPLEMENTARY MATERIALS

Supplementary material for this article is available at http://advances.sciencemag.org/cgi/content/full/6/10/eaaz1767/DC1 Fig. S1. Lung parenchymal immune cell profiles following vaccination. Fig. S2. FACS gating strategy to identify tetramer^+^ CD4^+^ T_RM_. Fig. S3. Vaccine empirical integrated model. Table S1. Health assessment scoring criteria. Table S2. Raw data for VEIM.

## Appendix

View/request a protocol for this paper from *Bio-protocol*.
