# Supplementary material for: A systematic approach to simultaneously evaluate safety, immunogenicity, and efficacy of novel tuberculosis vaccination strategies
Source: Sci Adv. 2020 Mar 4;6(10):eaaz1767. doi: 10.1126/sciadv.aaz1767 (PMC7056300; doi:10.1126/sciadv.aaz1767)
Supplement: http://advances.sciencemag.org/cgi/content/full/6/10/eaaz1767/DC1 [file supp_6_10_eaaz1767__index.html]

Science Advances | Science AdvancesAAASSearchScience AdvancesMenu

## Supplementary Materials

**This PDF file includes:**

- Fig. S1. Lung parenchymal immune cell profiles following vaccination.
- Fig. S2. FACS gating strategy to identify tetramer+ CD4+ TRM.
- Fig. S3. Vaccine empirical integrated model.
- Table S1. Health assessment scoring criteria.
- Table S2. Raw data for VEIM.

Download PDF

**Files in this Data Supplement:**

- Adobe PDF - aaz1767\_SM.pdf
