# Supplementary material for: A systematic approach to simultaneously evaluate safety, immunogenicity, and efficacy of novel tuberculosis vaccination strategies
Source: Sci Adv. 2020 Mar 4;6(10):eaaz1767. doi: 10.1126/sciadv.aaz1767 (PMC7056300; doi:10.1126/sciadv.aaz1767)
Supplement: Download PDF [file aaz1767_SM.pdf]

Science Advances
AAAS

advances.sciencemag.org/cgi/content/full/6/10/eaaz1767/DC1

Supplementary Materials for

A systematic approach to simultaneously evaluate safety, immunogenicity, and efficacy of novel tuberculosis vaccination strategies

Visai Muruganandah, Harindra D. Sathkumara, Saparna Pai, Catherine M. Rush, Roland Brosch, Ashley J. Waardenberg, Andreas Kupz*

*Corresponding author. Email: andreas.kupz@jcu.edu.au

Published 4 March 2020, *Sci. Adv.* **6**, eaaz1767 (2020)
DOI: 10.1126/sciadv.aaz1767

This PDF file includes:

Fig. S1. Lung parenchymal immune cell profiles following vaccination.
Fig. S2. FACS gating strategy to identify tetramer^{+} CD4^{+} T_{RM}.
Fig. S3. Vaccine empirical integrated model.
Table S1. Health assessment scoring criteria.
Table S2. Raw data for VEIM.

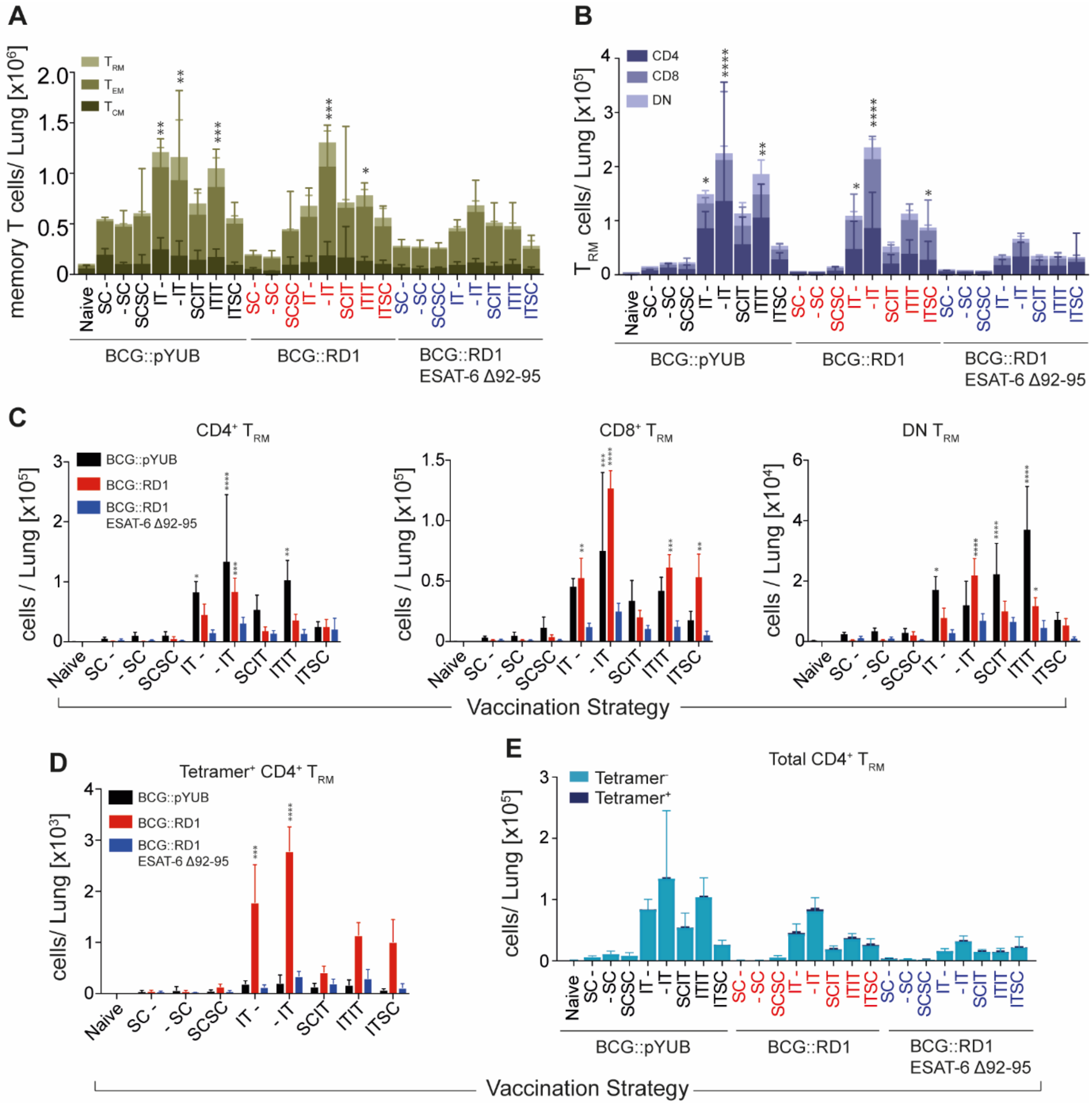

Fig. S1. Lung parenchymal immune cell profiles following vaccination. 60 days following prime vaccination, animals were sacrificed and perfused lung tissue was harvested for FACS analysis. (**A**) Number of $CD44^{hi}$ T lymphocytes T_{CM}, T_{EM} and T_{RM}. Indicated significance is of total memory T lymphocyte numbers. (**B**) Number of $CD4^+$, $CD8^+$ and DN T_{RM}. Indicated significance is of total T_{RM} numbers. (**C**) Total numbers of $CD4^+$, $CD8^+$ and DN T_{RM}. (**D**) Total numbers of tetramer$^+$ T_{RM}. Proportion of tetramer$^+$ and tetramer$^-$ $CD4^+$ T_{RM}. (**E**) Tetramer$^+$ and tetramer$^-$ cells amongst total $CD4^+$ T_{RM}. (A-D) Results are presented as mean values ± SEM from two pooled independent experiments (n = 5-10 mice per group). The statistical significance of differences between each experimental group relative to unvaccinated controls is shown. *, $p < 0.05$; **, $p < 0.01$; ***, $p < 0.001$; ****, $p < 0.0001$. The p values were determined using a one-way ANOVA followed by a Dunnett's multiple comparison test.

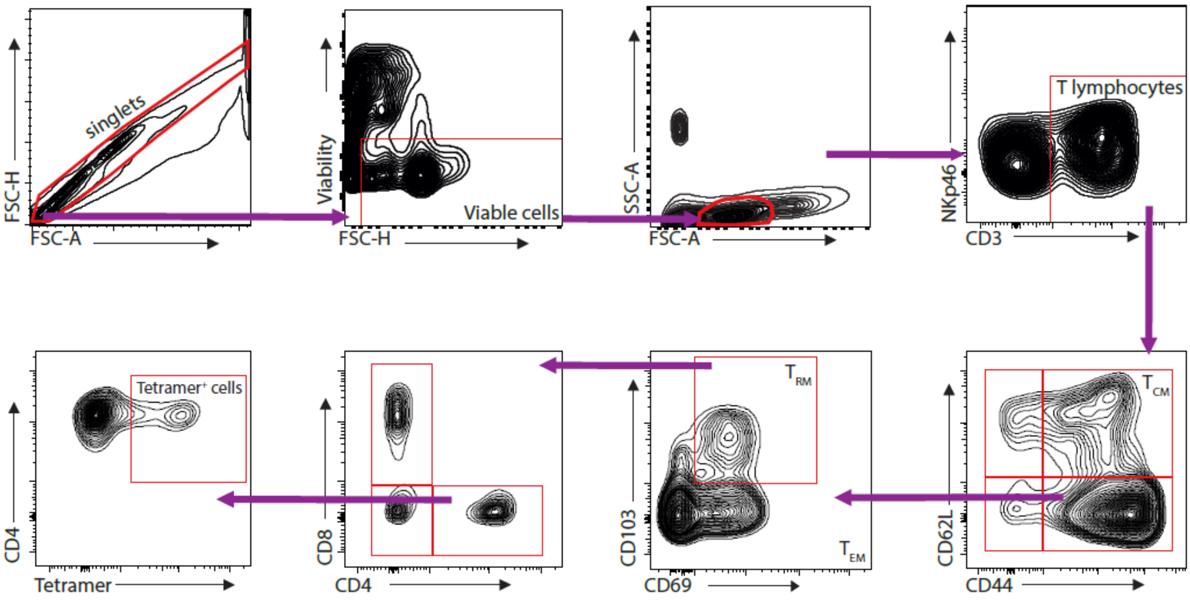

Fig. S2. FACS gating strategy to identify tetramer$^+$ CD4$^+$ T$_{RM}$. Lung and BALF cells were incubated with viability stain, incubated with the tetramer and monoclonal antibodies. Tetramer$^+$ cells were identified amongst CD3$^+$CD44$^+$CD62L$^-$CD69$^+$CD103$^+$CD4$^+$ cells.

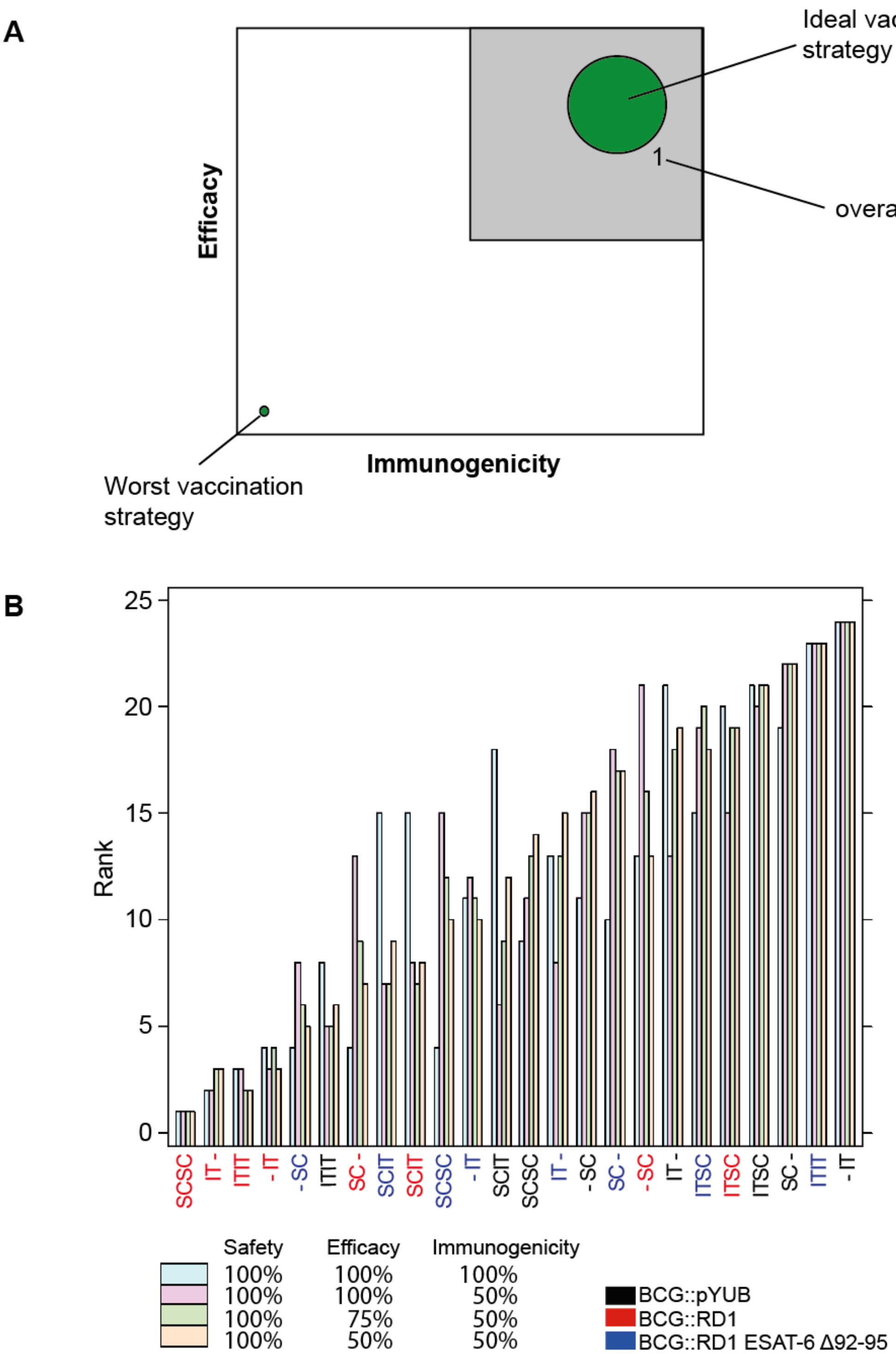

Fig. S3. Vaccine empirical integrated model. (**A**) Schematic illustration of how to interpret the VEIM plot shown in Fig. 6D. The ideal vaccination strategy is represented as a large circle in the top right quadrant, the worst vaccination strategy is represented by a small circle in the bottom left quadrant. The number next to the circle shows the overall rank of the vaccination strategy. (**B**) Four scenarios for differential weighting of VEIM parameters. The best strategy has lowest overall rank on y-axis. Blue: safety, efficacy and immunogenicity are equally weighted; Purple: safety and efficacy are equally ranked, and then immunogenicity weighted least (50% or penalty x 2); Green: safety is highest ranked, followed by efficacy (75% or penalty x 1.5), and then immunogenicity weighted least (50% or penalty x 2); Orange: safety is highest ranked, followed by efficacy (50% or penalty x 2) and then immunogenicity weighted least (50% or penalty x 2).

Table S1. Health assessment scoring criteria.

Score	3	2	1	0
Appearance	Shiny and well groomed fur, clear eyes, normal posture	Untended fur, clear eyes, normal posture	Untended fur, discharge from eyes or nose or slight hunching	Scruffy fur, definite hunching
Behaviour	Normal social interactions with other mice. Behaviours such as foraging, feeding, nesting and burrowing readily observed.	Normal social interactions with other mice. Behaviours such as foraging, feeding, nesting and burrowing observed.	Little interactions with other mice. Behaviours such as foraging, feeding, nesting and burrowing barely observed.	Avoidant behaviour, no interaction with other mice. Behaviours such as foraging, feeding, nesting and burrowing not observed.
Movement	Active	Less active	Barely active	Inactive
Response to stimuli	Instantaneous	Slightly delayed	Delayed	Significantly delayed
Breathing	Physiological	Physiological	Tachypnoea	Dyspnoea
Weight loss	Normal weight changes that reflect normal growth patterns	Less than 10%	10% - 15%	More than 15%

Instruction:

- Animals that were assigned a score of 1 were monitored twice daily
- Animals that were assigned a score of 0 were euthanized immediately

Caption: Subjective clinical health score criteria incorporating several observations. The lowest health score assigned during the course of a week was considered the overall score for that particular week.

Table S2. Raw data for VEIM.

	Efficacy			Safety									Immunogenicity							Ranking			
	Lung_CFU	Histopath.	Overall_Score	Clin._Score	Weight_loss	Lung_CFU	Spleen_CFU	Histopath.	IL1beta	IL6	TNFa	Overall_Score	BALF_TRM	LUNG_TRM	IL2	IL12p70	IFNg	IgA	Overall_Score	Efficacy	Safety	Immuno	Overall_Score
**RD1-SCSC**	2.700064104	1.76214908	4.462213187	-0.42857143	-1.46690821	-0.107234	-0.9010166	-0.0129208	-1.6395365	-0.9810429	-0.3593529	-3.909961923	1.60040343	1.51166041	1.53244251	0.84149677	-0.1900054	2.63603664	6.476078443	4	9	8	21
**RD1-IT-**	2.676999942	1.3020266	3.97902654	0	-1.29556333	-1.1350958	-1.0230239	-0.5157783	-0.0256383	-0.1013284	-0.0453266	-4.026892441	2.92630456	2.60848477	0.00689161	0.326849	-0.2390005	1.71797253	7.284341895	10	10	5	25
**RD1-ITIT**	2.762828003	1.42416675	4.186994757	-1	-1.48961901	-1.3436285	-1.0695304	-1.442099	-0.1647237	-0.0024801	-0.1032888	-6.435041142	3.03122425	2.76072444	0	-0.0780696	-0.1577403	2.11752055	7.830865925	6	19	3	28
**ESAT-SCSC**	2.624324237	1.7850544	4.409378634	0	-1.2398002	-0.107234	-0.8246404	-0.0170128	-0.0533427	-0.1895478	0.07393663	-2.245005388	0.92897904	0.93009308	0.3812311	0.30670201	-0.2442585	0.09466883	2.101632477	5	1	24	30
**RD1-SC-**	3.025489958	1.86101306	4.886503015	0	-2.60042051	-0.214468	-0.7535983	-0.0222833	-0.0546575	-0.0644817	-0.0189555	-3.63680175	1.18081318	0.86754407	0	0.04784911	-0.1450733	0.49078188	2.506731063	1	6	23	30
**ESAT--SC**	3.181045557	1.43983873	4.620884289	0	-2.82347302	-0.107234	-0.8003429	-0.0155543	-0.0189705	-0.2434007	0.10581936	-3.798788133	1.34962705	1.1353726	0.19359147	0.10109474	0.98014107	0.82869977	3.738641851	3	8	19	30
**RD1--IT**	2.947000907	1.03228239	3.979283294	0	-3.63761467	-1.0229687	-1.0639399	-0.9947696	-0.1177671	-0.1335692	-0.2932396	-6.900818164	3.27721755	3.29090461	0.29727881	-0.0780696	0.00549701	2.48356053	9.126584756	9	20	1	30
**pYUB-ITIT**	2.951191285	0.63046427	3.581655558	-0.14285714	-0.69332155	-1.2675076	-1.0293369	-1.5205914	-0.0668662	-0.1179802	0.02542209	-4.706755982	2.57526205	3.14598207	0.00689161	0.42074839	0.20434543	2.54226618	8.474172103	18	13	2	33
**pYUB-SCSC**	2.681166296	1.04021227	3.721378562	-0.42857143	-1.06414635	-0.1290878	-0.969961	-0.0119858	-0.7066029	-0.7418938	-0.4520031	-3.237252283	0.97202832	1.99555529	0.53535246	1.05124137	0.19287341	1.09366193	4.654401288	14	4	16	34
**ESAT-SC-**	2.406386255	1.44581478	3.852201036	0	-1.49631058	-0.2939637	-0.8951341	-0.0195365	-0.4372598	-0.135695	0.04388349	-2.88130207	0.89980514	1.12557377	0.29070046	0.12358022	-0.2315915	1.00714506	3.093420373	12	2	21	35
**pYUB--SC**	2.356713406	1.41203399	3.768747397	0	-2.60042051	-0.053617	-0.920084	-0.0178841	-0.0542818	-0.4109822	-0.0504811	-3.763920716	1.34657505	1.88222156	0.00689161	1.27753529	0.20864744	0.70062344	4.427111488	13	7	17	37
**ESAT--IT**	2.514222941	1.51833901	4.032561955	0	-2.41082587	-1.1332526	-1.0789459	-0.4059307	-0.5237539	-0.1399465	0.34649858	-5.134689066	2.36789165	2.34859648	0	-0.0629594	-0.2870396	0.64657878	5.246400588	8	15	14	37
**RD1--SC**	2.789934813	2.02247941	4.812414225	0	-3.27143681	-0.3851583	-0.7882544	-0.0953256	-0.1296941	-0.8988465	0.07114913	-4.859305633	1.38057941	0.6918636	0	0.10846998	0.44095593	0.69752788	2.953112855	2	14	22	38
**ESAT-IT-**	2.49121017	1.18733737	3.678547535	0	-1.61898946	-1.0533173	-0.9902645	-0.6425057	-0.0027235	-0.0403896	0.13729966	-4.273681375	2.26157306	2.11571122	0	-0.0780696	-0.2750896	1.64213976	5.90170432	16	11	11	38
**ESAT-ITSC**	2.34810046	1.31612244	3.664222902	0	-1.59668421	-0.7450151	-0.835425	-0.194333	-0.0122087	-0.0226749	0.21021118	-3.313014793	1.78951343	1.55277717	0	-0.0478491	-0.2397175	0.8520705	4.098505559	17	5	18	40
**RD1-SCIT**	2.801031736	1.26935553	4.070387267	-2	-3.35879904	-1.3667876	-1.1250897	-1.1318511	-0.8612779	-1.375019	-0.2390939	-9.807657688	2.90702125	2.31170063	0.04260265	-0.0780696	-0.2153395	1.04679709	6.181916836	7	24	9	40
**ESAT-SCIT**	2.47727782	1.3892873	3.866565116	-1.57142857	-3.49263054	-1.0595038	-1.0499457	-0.6036126	-0.0750366	-0.0910538	-0.2841572	-7.927203769	2.34059191	2.15526296	0.62682287	0.31245829	-0.1575013	2.17370728	6.930155419	11	22	7	40
**pYUB-SCIT**	2.753549744	0.92596194	3.679511687	-2	-3.67107255	-1.0840256	-0.8539349	-1.4879632	-0.2885013	-0.3004422	-0.4080066	-9.429312975	2.02274006	2.55782435	0.78940211	1.41406715	0.32288968	2.38921968	7.811903741	15	23	4	42
**pYUB-SC-**	2.442302415	1.0599317	3.502234114	0	-1.68590522	-0.214468	-0.8908875	-0.021837	-0.4171624	-0.1707702	-0.1655988	-3.064274909	0.93547701	1.739598	0	0.75893007	-0.0872352	0.78263768	3.681610977	20	3	20	43
**RD1-ITSC**	2.347509252	1.12921755	3.476726807	-0.14285714	-1.64129472	-1.0251178	-1.0275972	-0.7343559	-0.0216939	-0.0761734	0.0636417	-4.582631197	2.39582169	2.2931351	0.03101223	0.07465181	-0.2151005	1.40228577	6.054763752	22	12	10	44
**pYUB-ITSC**	2.574559206	1.00554652	3.580105723	0	-1.61898946	-1.2443827	-1.1371753	-0.963462	-0.2771378	-0.7500426	-0.2734793	-5.397562719	1.72712572	2.43215176	0.06922931	0.52598045	0.51337308	1.11693114	5.645736234	19	16	12	47
**pYUB-IT-**	3.130238074	0.10918737	3.239425439	0	-1.53441539	-1.3905137	-1.2435227	-1.3279508	-0.0965428	-0.560849	-0.35678	-5.834459883	2.16334544	3.07580871	0.21050723	1.01688355	0.22537748	1.40453348	7.127943712	23	18	6	47
**ESAT-ITIT**	2.333122876	1.16622561	3.499348482	-0.14285714	-2.75655726	-1.1076512	-1.0498794	-0.5734587	-0.014087	0.10841428	0.25364724	-5.514412233	2.13754674	1.82004852	0	-0.0235648	-0.1943074	1.27853922	5.163510412	21	17	15	53
**pYUB--IT**	2.316331051	0.91630412	3.232635171	0	-3.24727278	-1.3805293	-1.0157689	-1.8162192	-0.106028	-0.0467669	0.13988132	-7.464094691	1.7200855	2.54531538	0.01503623	0.33926099	0.25883755	1.06113154	5.530910677	24	21	13	58
